# Understanding entrance‐air‐kerma as a quality‐control metric for dual‐energy x‐ray absorptiometry

**DOI:** 10.1002/acm2.13779

**Published:** 2022-09-04

**Authors:** Matthew Allan Thomas, Jorge Ernesto Jimenez, Samuel John Fahrenholtz, Khushnood Hamdani, William Daniel Erwin

**Affiliations:** ^1^ Department of Imaging Physics UT MD Anderson Cancer Center Houston Texas USA; ^2^ Section of Medical Physics, Department of Radiology Mayo Clinic Arizona Phoenix Arizona USA

**Keywords:** bone mineral density, dual‐energy x‐ray absorptiometry, entrance‐air‐kerma, x‐ray absorptiometry

## Abstract

**Purpose:**

The low exposures, unique x‐ray beam geometry, and scanning design in dual‐energy x‐ray absorptiometry (DXA) make measurement and quality‐control strategies different from traditional x‐ray equipment. This study examines the dependence of measured entrance‐air‐kerma (EAK) on both dose sensor type and scan length. The feasibility of using EAK to compare scanner output between different scan modes, individual scanners, and scanner platforms was also established. Finally, the congruence between measured and vendor‐reported EAK was analyzed.

**Methods:**

Four Hologic DXA scanners at two institutions and all four available scan modes were tested. EAK was measured directly by three types of Radcal dose sensors: 60‐cc pancake ion‐chamber (IC), 180‐cc pancake IC, and solid‐state detector. The coefficient of variation (COV) was used to assess the dependence of EAK on scan length. Variations in EAK between the types of dose sensors as well as measured versus vendor‐reported values were evaluated using Bland–Altman analysis: mean ±95% prediction interval (PI): 1.96*σ*.

**Results:**

Dose sensor variations in EAK were minimal, with a −3.5 ± 3.5% (mean ±95% PI) percent difference between the two sizes of IC's. The solid‐state detector produced highly similar measurements to the 180‐cc IC. These small differences were consistent across all scanners and all scan modes tested. Neither measured nor vendor‐reported EAK values were found to show relevant dependence on scan length, with all COV values ≤4%. Differences between measured and reported EAK were higher at −6 ± 48%. Likely errors in vendor‐reported EAK calculations were also identified.

**Conclusion:**

It is feasible to quantify DXA scanner stability using EAK as a quality‐control metric with a variety of solid‐state and IC dose sensors, and the scan length used is not critical. Although vendor‐reported EAK was consistent among scanners of the same platform, measured EAK varied significantly from scanner to scanner. As a result, measured and reported EAK may not always be comparable.

## 1 INTRODUCTION

Bone mineral density (BMD) assessments are an important part of patient diagnosis and care with respect to osteoporosis and other bone diseases.^1^, ^2^, ^3^ Evaluation of treatments in clinical trials also relies heavily on consistent BMD data to track patient progress and outcomes.^4^ The current clinical standard for areal BMD analysis is dual‐energy x‐ray absorptiometry (DXA).^1^, ^2^, ^3^, ^5^ DXA is a low exposure x‐ray imaging technique that differentiates between bone and soft tissue by the use of two different x‐ray energies.^5^ The first generation of x‐ray based DXA scanners suffered from very long scan times. They utilized a pinhole collimator that created a pencil x‐ray beam coupled to a single detector.^5^ This necessitated a two‐dimensional raster scan across the object of interest, which was a very slow process (5 to 10 min per scan). Modern DXA scanners are an order of magnitude faster due to the use of slit collimators that produce thin x‐ray fan beams coupled to linear detector arrays spanning the full width of the scan area.^5^ This enables a single dimension scanning technique only along the length of the scan area that significantly reduces scan time.

Two strategies exist to achieve dual‐energy imaging in DXA. The first involves using a K‐absorption edge filter to separate the poly‐energetic x‐ray beam into components with low and high energies.^5^ This method attempts to mimic the emissions from ^153^Gd, as used in the original radionuclide‐based dual‐photon absorptiometry method of measuring areal BMD.^5^ The second DXA strategy involves fast primary voltage (kVp) switching to create low and high energy x‐rays directly at the source.^5^ This method produces a wider x‐ray beam spectrum, so beam hardening issues must be actively addressed to enable consistent BMD measurement. Hologic DXA scanners implement the kVp switching technique but also require a rotating calibration wheel containing bone and soft tissue equivalent filters to handle beam hardening. The filters in the calibration wheel actively provide a standard bone signal reference, enabling calibration of the DXA image on a pixel‐by‐pixel basis.^5^, ^6^ Because the kVp switching method does not require energy discrimination at the detector with pulse height analysis, it offers a much wider dynamic range with respect to x‐ray energies detected and utilized for imaging.

Raw BMD values provided by a specific DXA scanner must be used with caution for clinical assessment due to differences in DXA scanner performance, scanning parameters and patterns, beam geometry, and algorithms across vendors.^2^ Tracking longitudinal changes in BMD for a specific patient is acceptable, but results should generally not be compared between different scanners, unless valid conversion formulas have been established. In particular, any scanner used must be providing BMD values with sufficient precision, as assessed with quality‐control (QC) metrics.^2^ In order to avoid such complications, T‐score is used for evaluations related to osteoporosis—evaluating fracture risk in adults aged over 50 years. T‐score is the number of standard deviations the BMD value of interest deviates from the mean of a population of young, Caucasian women.^7^ In contrast to T‐score, Z‐score is the number of standard deviations away from the mean for BMD from a population matched group in terms of gender, age, and ethnicity.^2^ Z‐score is typically used for bone mass assessments in children and young adults with the hope of identifying possible bone pathology.

Two aspects of BMD measurements analyzed for its clinical performance are precision and accuracy. Clinical precision of DXA is measured by least significant change, which determines whether a longitudinal change in areal BMD is physiological or measurement error. Areal BMD precision using DXA with respect to different vendors and repeat measurements is high, with values <2% absolute error typically determined.^2^, ^3^, ^8^, ^9^ However, some vendors may show higher errors than others.^8^, ^9^ Areal BMD accuracy using DXA is more difficult to evaluate explicitly but is usually determined to be much lower than precision at ≤7% absolute error.^2^ Based on how BMD is used clinically, accuracy is less relevant than precision because changes in BMD over time are typically tracked for clinical decisions.

Possibly due to the inherently low x‐ray exposures relative to traditional x‐ray imaging, required regulatory compliance and QC standards are less comprehensive for DXA systems. As a result, there is much less previous work related to equipment quality assurance, commissioning, and QC for DXA than many other imaging modalities. The International Society for Clinical Densitometry provides accreditation of systems and also offers standards for QC, precision, and so forth.^1^ Sheahan and colleagues outlined relevant quality assurance and commissioning tests for DXA in two previous works.^10^, ^11^ In their studies, Sheahan et al. included dose‐area‐product (DAP) in their list of recommended measurements and utilized an 1800 cubic centimeter (cc) ion chamber as the dose sensor. An even older study evaluated measuring DAP directly using x‐ray film.^12^ Most previous studies have focused on assessments of internal precision error in BMD from DXA related to both equipment performance and technologist repeatability.^1^, ^13^

The low exposures, unique x‐ray beam geometry, and scanning design in DXA make measurement and QC strategies different compared to traditional x‐ray equipment. Daily or weekly measurements that evaluate BMD precision using vendor‐supplied phantoms are considered the most relevant and critical aspect of QC for DXA.^1^, ^13^ More exhaustive measurements related to x‐ray tube output are not straightforward. In fact, in our discussions with technical personnel from Hologic, it was suggested that measurement of entrance skin dose and/or DAP is difficult due to the following: brass filtration in the high energy beam, the pulsed nature of the source, periodic appearance of reference materials in and out of the beam, and the alternating high and low energy pulses. DXA is the current clinical standard for areal BMD assessments, making DXA scanners a very common part of equipment fleets in a variety of diagnostic imaging and other medical practices. Total body DXA scans are also employed for clinical applications beyond BMD, such as body composition.^14^, ^15^ Therefore, as the clinical use of DXA continues to grow, it is important to establish validated methods to ensure equipment quality assurance and QC programs can be carried out consistently and effectively. In fact, an AAPM task group (TG 367) was recently formed to develop recommendations for QC of DXA scanners.

The current ACR/AAPM medical physics technical standard for DXA outlines several recommended tests for equipment performance evaluation, two of which are directly related to the current study: (1) measurement of entrance‐air‐kerma (EAK) for the most common clinical procedures, and (2) verification of displayed dose and radiation output metrics (if applicable).^16^ Considering the low doses at which DXA systems operate, measurements can be challenging and/or labor intensive, with some previous work even utilizing thermoluminescent dosimeters.^17^ The utilization of DAP, which is strongly related to EAK, has been suggested previously as a metric for DXA QC protocols.^10^, ^11^, ^18^ However, we are not aware of any recent studies comparing different measurement strategies and analyses using modern equipment. In this study, the dependence of measured EAK on both dose sensor type and scan length was examined. The feasibility of using EAK to compare scanner output between different scan modes, individual scanners, and scanner platforms was also established. Finally, the congruence between measured and vendor‐reported EAK was analyzed to understand potential sources of differences between measured and vendor‐reported EAK.

## 2 MATERIALS AND METHODS

### 2.1 DXA scanners tested

Four different DXA scanners were assessed in this study, all from Hologic (Bedford, MA, USA). Table 1 outlines the scanners’ key radiographic specifications and attributes. There were some distinctions between the different systems, including number of detector channels, scintillator material, and x‐ray beam filtration. The nominal entrance skin dose values were obtained from the vendor's specifications documentation, and they were equivalent for Horizon and Discovery systems. Note that no entrance skin dose data wer available for High Definition (HD) mode.

**TABLE 1:** Information and specifications for the four DXA scanners tested in this study

	**Horizon 1**	**Horizon 2**	**Horizon 3**	**Discovery**
Model	Horizon A	Horizon W	Horizon A	Discovery A
Location	MD Anderson (Main)	MD Anderson (Satellite)	Mayo Clinic, Phoenix, AZ	MD Anderson (Main)
Detector	216 channel (2 mm)	128 channel (2 mm)	216 channel (2 mm)	216 channel (2 mm)
Detector material	GOS	GOS	GOS	CdWO_4_
SID (cm)	107.2 ± 1.2	107.2 ± 1.2	107.2 ± 1.2	107.0 ± 0.8
SOD (cm)*	42.3 ± 0.8	42.3 ± 0.8	42.3 ± 0.8	42.4 ± 0.6
HVL (mm Al)	100 kV: 4.4	100 kV: 4.4	100 kV: 4.4	100 kV: 5.0
	140 kV: 6.0	140 kV: 6.0	140 kV: 6.0	140 kV: 6.5
X‐ray tube age^#^	22 months	20 months	33 months	25 months
Nominal Entrance Skin Dose ( μ Gy)				
AP Spine (Array)	130	130	130	130
AP Spine (Fast)	70	70	70	70
AP Spine (Express)	40	40	40	40
Whole body (Array)	8	8	8	8

Abbreviations: CdWO_4_, cadmium tungstate; GOS, gadolinium oxysulfide; HD, High Definition mode; HVL, half value layer; SID, source to image (detector) distance; SOD, source to object distance.

*Specified from source to patient skin surface.

^#^ All but Discovery were original x‐ray tubes from scanner installation.

### 2.2 Scan acquisitions and EAK measurements

EAK was measured for all four available scan modes on all four scanners: HD, Array, Fast Array (Fast), and Express. All measurements were made using the AP Spine acquisition, but at different scan lengths as provided by the scanner: 1, 2, 4, 5, 6, 7, and 8 inches (2.5 to 20.3 cm). Array mode is exclusively used clinically at our institutions, with the lumbar‐spine being the most common skeletal region for areal BMD assessment. The four scan modes tested differed with respect to both scan speed and beam collimation. Table 2 provides a detailed comparison of the acquisition characteristics for each scan mode.

**TABLE 2:** Scan mode parameters for the DXA scanners tested

	**HD**	**Array**	**Fast**	**Express**
Scan speed (mm/s)	1.2	2.5	5.0	13.7
Horizon beam collimation (mm)	47.2 × 0.4	47.2 × 0.8	47.2 × 0.8	47.2 × 1.6
Discovery beam collimation (mm)	61.0 × 0.5	61.0 × 1.0	61.0 × 1.0	61.0 × 2.0
Exposure (mAs)*	306	154	77	28

Abbreviation: HD, High Definition mode.

*For a 6‐inch (15.2 cm) AP Spine acquisition.

A Radcal (Monrovia, CA, USA) 10×6‐60 60‐cc pancake ion chamber, 10×6‐180 180‐cc pancake ion chamber, and AGMS‐DM+ solid‐state detector (SSD) were used for the EAK measurements. A Radcal Accu‐Gold+ digitizer/electrometer and associated software were used to analyze the measurements. Table 3 outlines the various specifications of the three dose sensors employed. Each sensor was placed on top of the scanner table (with 1 inch cushion in place) and scanned for the desired length. The center of the sensor was placed in the center of the scan region both laterally and in the direction of the x‐ray tube/detector c‐arm motion. Due to the low exposures involved, low trigger mode on the Accu‐Gold+ software was used. The 180‐cc ion chamber was used for all scan modes and scan lengths from 4 to 8 inches. The 60‐cc ion chamber was used for all scan modes and scan lengths of 4, 5, 6, and sometimes 7 inches (due to the smaller size of the 60‐cc detector, for slower scan modes at 7 inches and all scan modes at 8 inches it could not be used due to the EAK acquisition timing out from lack of signal readable by the digitizer). For most scan modes and scan lengths, the ion chambers could still acquire signal even when the scan range was outside of their active area. This was likely due to scatter contributions from the scanner table. The solid‐state detector could only be used with the two shortest (1‐ and 2‐inch) scan lengths due to its much smaller active area and the measurement timing out from lack of signal more readily than with the ion chambers. Table 4 shows the combinations of dose sensors, scan modes, and scan lengths used for the analysis of each scanner.

**TABLE 3:** Specifications for the Radcal dose sensors used in this study

	**60‐cc IC**	**180‐cc IC**	**SSD**
Model	10×6‐60	10×6‐180	AGMS‐DM+
Height (cm)	1.3	2.3	3.6 × 2.0 × 1.2
Diameter (cm)	9.2	11.8	‒
Active volume (cm3)	60	180	‒
Accuracy	±4%*	±4%*	±5%
Min dose (nGy)	10	2	80
Min dose rate (nGy/s)	2	1	80

Abbreviations: IC, ion chamber; SSD, solid state detector.

*At 150 kVp and 10.2 mm Al half‐value layer.

**TABLE 4:** Dose sensor, scan mode, and scan length combinations tested in this study

	**Horizon 1**	**Horizon 2**	**Horizon 3**	**Discovery**
Dose sensors used	60‐cc IC, 180‐cc IC, SSD	60‐cc IC, 180‐cc IC, SSD	60‐cc IC	60‐cc IC, 180‐cc IC, SSD
Scan modes tested	HD, array, fast, express	HD, array, fast, express	HD, array, fast, express	HD, array, fast, express
Scan lengths tested (inches)*	1, 2, 4, 5, 6, 7, 8	1, 2, 4, 5, 6, 7, 8	4, 5, 6, 7, 8	1, 2, 4, 5, 6, 7, 8

Abbreviations: HD, high definition; IC, ion chamber; SSD, solid state detector.

*1‐ and 2‐inch scans only tested with SSD; 8‐inch scans only tested with 180‐cc IC.

EAK with backscatter was also assessed by placing 14 cm × 14 cm acrylic plates in total thicknesses from 4 to 8 inches on top of the measurement device to serve as a patient‐mimicking phantom. The backscatter assessments were only undertaken at one clinical site—not all sites associated with the measurements in this study had acrylic plates available. The majority of the EAK values reported in this study were without the acrylic phantom in place—those measurements made with backscatter included are identified specifically for clarification.

For each individual DXA scanner assessed, all measurements were achieved within one or two days to minimize x‐ray tube output changes between measurements with different sensors. All measurements were repeated at least twice and averaged. However, the repeatability of the dosimetric measurements was observed to be excellent in general (<1% change between repeat measurements in most cases). HD scan mode provided the most significant inconsistencies, though they were, at worst, comparable to the quoted accuracy of the sensors (Table 3).

Hologic reports DAP for each individual scan rather than EAK or entrance skin dose. Vendor‐reported DAP values were extracted from the DICOM header using the (0018,015e) DICOM tag (unit: cGy·cm2). We were unable to confirm the details of how the vendor determines their reported DAP measurements, as their methods were described as “proprietary.” We believe the reported DAP values come from a complex calculation involving many variables related to x‐ray technique parameters and equipment design, including “scan mode, x‐ray aperture, x‐ray mode, and other assumptions,” according to Hologic technical personnel. To compare measured values (EAK) and vendor‐reported values (DAP), scan field sizes were evaluated using scanned computed radiography (CR) plates (Fujifilm, Tokyo, Japan). The CR plates were scanned in the same way as the sensors, but separately. The plates were placed on the scanner table (with 1 inch cushion in place) to verify scan field sizes: scan lengths and beam widths at tabletop height with bed cushion in place, for all scan modes and scanners tested. The CR plates were then read and the scan length and width were derived from the CR images. It is worth noting that the vendor specifies a beam width of 4.5 inches (11.4 cm; at patient skin surface) for all scan modes and scanners tested. However, when calculating the beam geometry using the source‐to‐image distance (SID), source‐to‐object distance (SOD), and collimation information from the vendor specifications (Table 1), a beam width of 4.3 inches (10.9 cm) was obtained. This beam width matched much better with all CR plate measurements than 4.5 inches (11.4 cm) and, thus, was used in all calculations in this study.

Scan field size measurements with the CR plates validated the nominal sizes expected (2.5, 5.1, 10.2, 12.7, 15.2, 17.8, and 20.3 cm lengths; 10.9 cm width) within <2.5% absolute error for all scan modes and scanners assessed. Average deviations across all measurements were <1.5% absolute error. These values were comparable to the uncertainty in using CR plates for localization of radiation fields.^19^ As a result, all vendor‐reported DAP values were converted to EAK using nominal scan lengths and width as listed in detail above to ease comparisons across all scan modes and scanners. After conversion from DAP to EAK, vendor‐reported values could be compared directly to those measured with the dose sensors.

### 2.3 Quantitative analysis

EAK values were compared between the various scan modes and scanners. For both reported and measured EAK, coefficients of variation (COV) were calculated to determine the effect of scan length on EAK values:

$$\mathrm{COV}\left(\%\right)\;=\frac{\sigma }{\mu }\;\times 100,$$

where *σ* and *μ* are the standard deviation and mean, respectively, of the distribution of EAK values across all scan lengths for a given scanner and/or scan mode tested. The differences between readings of the three dose sensors were compared using measured EAK values from three of the four DXA scanners (Horizons 1 and 2, and Discovery) and Bland–Altman analysis.^20^ Measured and vendor‐reported EAK values were also compared with Bland–Altman analysis, with all measured EAK values coming from the 60‐cc ion chamber because it was the only device consistently used for all four DXA scanners assessed. In the Bland–Altman analyses, percent differences in the two sets of values compared were plotted as a function of the mean of the values compared. The percent differences were calculated as follows:

$$\%\;\mathrm{Difference}\;=\frac{\left(\mathrm{New}-\mathrm{Reference}\right)}{\mathrm{Mean}\;\mathrm{of}\;\mathrm{New}\;\mathrm{and}\;\mathrm{Reference}}\;\times 100$$

For the sensor comparisons, the 60‐cc ion chamber measurement served as the reference value. In the measured versus reported EAK comparisons, reported EAK served as the reference value. The comparisons were analyzed with the mean percent difference and the 95% prediction interval (PI, ±1.96*σ*) of the distribution of percent differences.^20^

## 3 RESULTS

### 3.1 Dependence of EAK on sensor type

**FIGURE 1:**
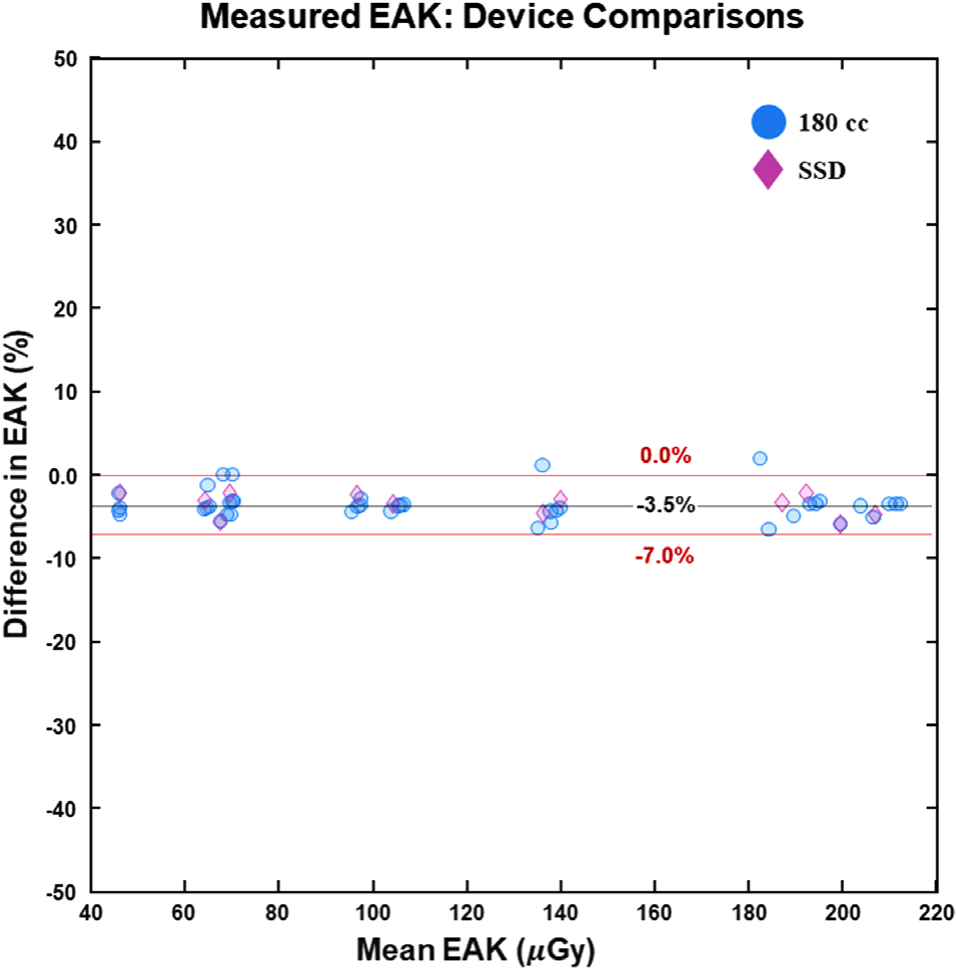
Bland–Altman plot comparing entrance‐air‐kerma measured with different sensors: 180‐cc ion chamber relative to the 60‐cc ion chamber (blue circles). The mean percent difference and 95% prediction interval range between measurements on the two sensors are included as dashed lines in the plots, along with their values. Data for the solid‐state detector (relative to those for the 60‐cc ion chamber (red diamonds) are also included for comparison

Figure 1 shows a Bland–Altman plot comparing EAK measurements between the two different ion chambers (180‐cc relative to 60‐cc). The values for the versus the 60‐cc chamber are also included for comparison. The 180‐cc chamber consistently produced slightly lower measurements, with a mean difference of −3.5% relative to those for the 60‐cc chamber. The solid‐state detector produced very similar measurements to those for the 180‐cc chamber. Overall, the measurements between sensors were highly consistent with a difference 95% PI range of approximately −7% to 0%.

**FIGURE 2:**
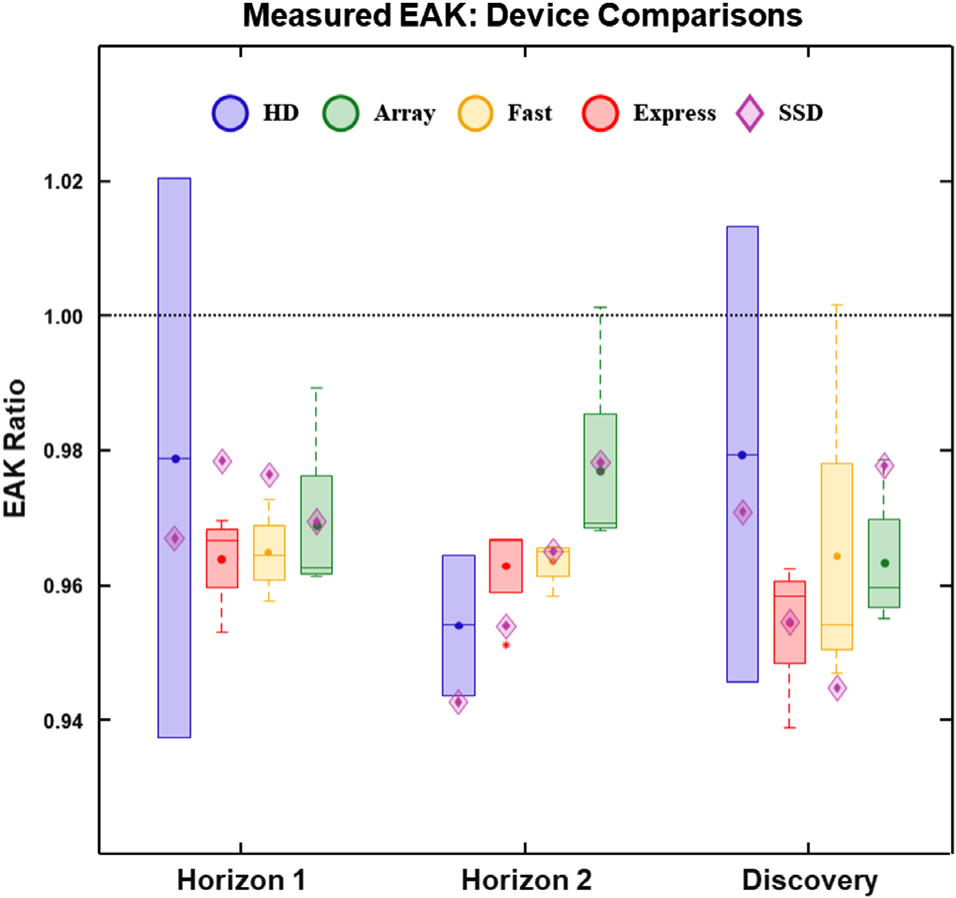
Boxplots of measured entrance‐air‐kerma ratios comparing the 60‐ and 180‐cc ion chamber measurements for all four scan modes on three different dual‐energy x‐ray absorptiometry scanners. The ratios are calculated relative to the 60‐cc measurement. Median values are shown with a horizontal line within each box, while the box represents the interquartile range (25th to 75th percentiles). Mean values are shown with a solid dot. The whiskers extend to 95% CI limits. Ratios for the solid‐state detector (relative to the 60‐cc ion chamber are also included for comparison (diamonds)

Figure 2 shows boxplots of measured EAK ratios for the 180‐cc ion chamber relative those for the 60‐cc chamber, broken down by both scan mode and three different DXA scanners (Horizons 1 and 2, and Discovery). The results were very similar to those in Figure 1 but they also showed that no specific scan mode or scanner deviated from the general trend. On average, the measured EAK differences between various types of sensors were small for all scan modes and scanners.

### 3.2 Dependence of EAK on scan length, scan mode, and platform

Table 5 includes vendor‐reported EAK values for all scanners and scan modes. COV for EAK is included in Table 5 to evaluate the effect of scan length on vendor‐reported values. Ratios are also included to more easily compare each scan mode to the clinical standard mode (Array), as well as compare Horizon and Discovery scanners. All reported EAK values for the Discovery system were higher than those for Horizon, but the amount of increase was not consistent across all scan modes. On the Discovery system, both the HD and Array modes had the same reported EAK values. However, on Horizon scanners the HD mode's reported EAK values were only 72% of those reported in Array mode. EAK COV was very low in general for reported values, with all scan modes for both scanner platforms showing <1% variation across scan lengths except for express mode on the Discovery scanner (COV ∼2%).

**TABLE 5:** EAK values and ratios for vendor‐reported data

	**Vendor reported**			
**Horizon**	**HD**	**Array**	**Fast**	**Express**
EAK (μGy)*	132 ± 0.1	182 ± 0.2	91 ± 0.4	69 ± 0.6
EAK COV^†^	0.11%	0.13%	0.42%	0.81%
Array ratio^#^	1.38	‒	2.00	2.64
Discovery ratio^&^	0.63	0.87	0.87	0.94
**Discovery**				
EAK	210 ± 0.2	209 ± 0.7	105 ± 0.4	73 ± 1.5
EAK COV	0.11%	0.36%	0.42%	2.05%
Array ratio	1.00	‒	1.99	2.86

Abbreviations: COV, coefficient of variation; EAK, entrance‐air‐kerma; HD, High Definition scan mode.

*EAK reported values derived from vendor‐reported DAP and nominal field size, values are given as: mean ± σ across all scan lengths.

^†^ EAK COV based on variation with scan length.

^#^ Ratio calculated from the mean EAK value of Array mode relative to the mean of each other scan mode.

^&^ Ratio calculated from the mean EAK value of each scan mode, relative to the Discovery scanner.

Table 6 shows the same type of data as Table 5, but for measured EAK values and with unique data for each of the three Horizon scanners. The measured values come from the 60‐cc ion chamber. EAK COV was higher for measured values, but still generally low. The HD scan mode had limited measured data for some scanners and therefore had more variability with a COV of ∼4% for three of the four scanners. Each of the three different Horizon scanners showed distinct measured EAK values, with ∼30% change in EAK between Horizon scanners with the lowest (#3) and highest (#2) measured values. Unlike the reported EAK values, the measured values showed that the Discovery scanner had the lowest EAK, being between 15% and 50% lower than the three Horizon scanners. For all four scanners, the measured EAK values were very similar between HD and Array modes. While the Discovery reported EAK matched with this observation, it was very different from the reported EAK for the Horizon systems.

**TABLE 6:** EAK values and ratios for measured data

	**Measured**			
**Horizon 1**	**HD**	**Array**	**Fast**	**Express**
EAK (μGy)*	185 ± 6.9	196 ± 1.8	98 ± 0.7	66 ± 0.5
EAK COV^†^	3.70%	0.90%	0.66%	0.69%
Array ratio^#^	1.06	‒	2.00	2.97
Discovery ratio^&^	1.34	1.39	1.40	1.40
**Horizon 2**				
EAK	206 ± 1.4	214 ± 1.9	107 ± 0.9	71 ± 0.4
EAK COV	0.69%	0.90%	0.86%	0.64%
Array ratio	1.04	‒	2.00	3.01
Discovery ratio	1.49	1.52	1.53	1.51
**Horizon 3**				
EAK	157 ± 5.1	160 ± 3.6	80 ± 0.4	56 ± 1.2
EAK COV	3.27%	2.26%	0.47%	2.10%
Array ratio	1.02	‒	2.00	2.86
Discovery ratio	1.14	1.13	1.14	1.19
**Discovery**				
EAK	138 ± 4.7	141 ± 1.4	70 ± 1.3	47 ± 0.4
EAK COV	3.42%	1.03%	1.86%	0.83%
Array ratio	1.02	‒	2.01	3.00

Abbreviations: COV, coefficient of variation; EAK, entrance‐air‐kerma; HD, High Definition scan mode.

*EAK measured values come from 60‐cc IC, values are given as: mean ± σ across all scan lengths.

^†^ EAK COV based on variation with scan length.

^#^ Ratio calculated from the mean EAK value of array mode relative to the mean of each other scan mode.

^&^ Ratio calculated from the mean EAK value of each scan mode, relative to the Discovery scanner.

### 3.3 Congruence between measured and vendor‐reported EAK

**FIGURE 3:**
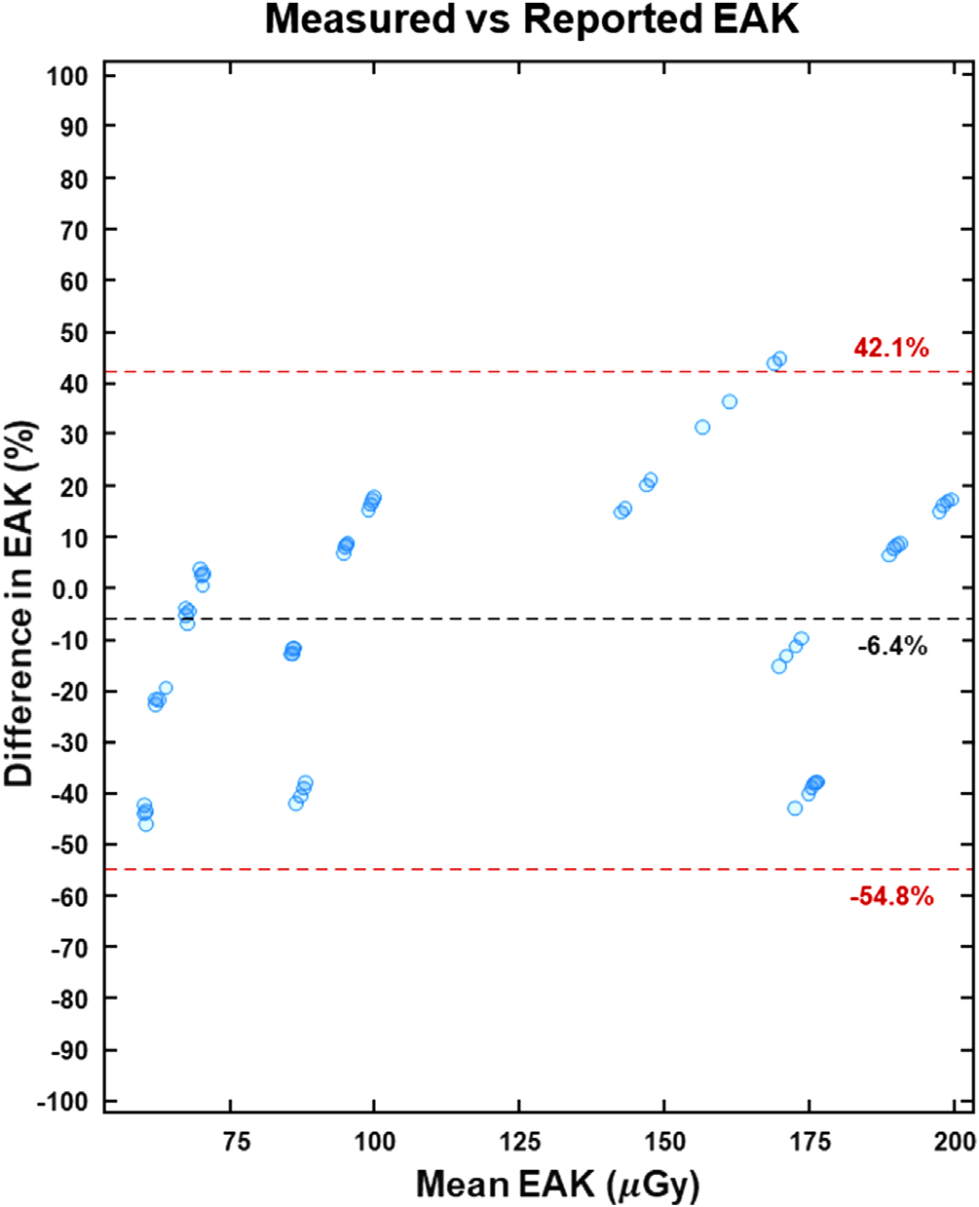
Bland–Altman plot comparing measured (60‐cc ion chamber) and reported entrance‐air‐kerma (EAK). The mean percent difference and 95% PI range between the EAK values are included as dashed lines in the plots, along with their values

The differences between measured and vendor‐reported EAK across all scan modes and scanners are shown in Figure 3 with a Bland–Altman plot. All measured EAK values were for the 60‐cc ion chamber. The mean percent difference between measured and reported EAK was approximately −6%. However, the variability between measured and reported EAK was high with a 95% PI range from −55% to +42%. These results indicate that a wide range of differences existed between measured and reported EAK across the various scan modes and DXA scanners assessed, with no clear or consistent bias. The EAK measurements that included backscatter, using the acrylic phantom, showed consistently increased values from 30% to 35% across various scan lengths and scan modes. Note that if all EAK measurements in this study were adjusted for backscatter, the mean percent difference between measured and reported EAK would adjust to +25% to +30%, making it clearly biased. These results suggest that vendor‐reported EAK is an in‐air value without any adjustment to include estimated backscatter.

**FIGURE 4:**
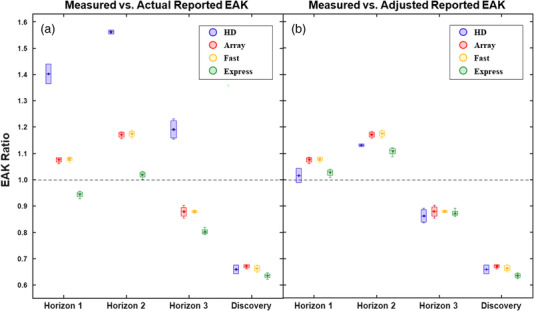
Boxplots of measured versus reported entrance‐air‐kerma (EAK) ratios for all four scan modes on four different dual‐energy x‐ray absorptiometry scanners: (a) unadjusted ratios, (b) ratios calculated using adjusted reported EAK values for HD and express scan modes. The ratios are calculated with the reported EAK value in the denominator. The boxplots are set up the same as in Figure 2

Figure 4 shows boxplots of ratios between measured and reported EAK, broken down by both scan mode and the four different DXA scanners. In Figure 4a, the ratios were calculated with the actual reported EAK value in the denominator. Overall, measured and reported EAK values were consistently different—similar to the data shown in Figure 3. More specifically, recall that all reported EAK values were the same among each of the three Horizon scanners. Therefore, the variations in the EAK ratios across the various Horizon scanners as seen in Figure 4a were due solely to different x‐ray tube outputs. The Discovery scanner showed the most comparable EAK ratios across scan mode, but also the largest consistent deviation between measured and reported EAK. All measured EAK values were much smaller than those reported for the Discovery scanner, with a >30% difference on average for all scan modes. As also seen in Figure 4a, EAK ratios for HD mode were very different between the Discovery and Horizon scanners. While the Discovery HD mode EAK ratios were similar to those for the other three modes, HD mode on the Horizon scanners had EAK ratios that were consistently higher than those for all the other three modes. These results make it clear that reported EAK values for HD mode maintained key fundamental differences between Horizon and Discovery scanners, much like the results in Table 5.

In Figure 4b, the ratios were re‐calculated after adjusting the reported EAK values for both HD and Express modes for the three Horizon scanners. The reported EAK values for HD and Express modes were adjusted so that they had the same relationships with the other scan modes as reflected with the Discovery scanner. As a result, reported EAK was increased by 38% for HD mode and decreased by 8% for Express mode. After these adjustments, all four scan modes showed much more consistent relationships between measured and reported EAK values on a particular scanner. The clearest deviations between measured and reported EAK transferred from being related to scan mode to the individual scanners instead.

## 4 DISCUSSION

In this study, EAK was measured on several different DXA scanners under various acquisition conditions. Different dose sensors were assessed and their measurements were compared to vendor‐reported EAK values that were derived from DAP and scan field size. To our knowledge, the distinctions between the various DXA scanners outlined in Table 1 played no known role in affecting the assessments that were the purpose of this study. As seen in Figures 1 and 2, two different ion chamber sizes produced consistent EAK measurements that were different from each other by only a few percent on average. A third set of measurements with the helped to further validate that none of the measurement devices showed erroneous behavior or clearly biased results. Table 6 shows that scan length had a minimal impact on measured EAK, with most COV values derived from variability with scan length being less than a few percent. The consistency in measured data with scan length would enable straightforward predictions of many lengths based only on a small sample of scan lengths/measurements.

Results from Figures 1 and 2 and Table 6 suggest that it is feasible to measure EAK on a DXA scanner using several different dose sensors, and scan length is not particularly critical. Although it may be preferable to use the largest ion chamber available (e.g., the 180‐cc pancake chamber as used in this study) and a scan length that is comparable to the chamber size, our results indicate that this is not necessary in order to obtain consistent EAK measurements. It would be reasonable to use a SS and a very short scan length to evaluate EAK and still obtain relevant measurements.

When assessing the results in Figure 4 as well as Table 5, it is clear that reported EAK for Horizon scanners was likely erroneous for HD and Express scan modes. For the Discovery scanner, HD and Array modes had the same reported EAK but on Horizon they were distinctly different. Also, when comparing measured to reported EAK, the Discovery scanner showed consistent ratios among all scan modes, even Express mode. When adjusting the reported EAK values for Horizon such that their relationships to Array mode matched better with Discovery (Figure 4b), the EAK ratios made much more sense. There were still clear changes in measured versus reported EAK among the individual scanners, but much less so among scan modes on a particular scanner. All of these results suggest that the reported EAK algorithm on Horizon scanners was incorrect for both HD and express modes. Most likely, the algorithms should be adjusted to fit the pattern among scan modes as seen on the Discovery scanner.

When a new Bland–Altman analysis was performed using the adjusted reported EAK values for Horizon HD and Express scan modes, the overall comparisons among measured and reported EAK were still poor. It is clear that the differences between measured and reported EAK stem mainly from different x‐ray tube outputs among individual scanners. The differences also do not appear to be due to backscatter because there was not a clear bias that would indicate a necessary adjustment for backscatter. Overall, our results suggest that the reported EAK values do not include backscatter. If we were to consistently include it in our measured EAK data, the ratios between measured and reported would all increase by 30%–35%. This would only serve to adjust the mean percent difference but would not affect the overall variability in the measured versus reported EAK values. Other work on DXA EAK (and DAP) has shown similar results for some vendors, where reported values appeared to not include any effects for backscatter in their calculation/algorithm.^10^, ^11^

Among our results in this study, the Discovery scanner showed lower measured EAK (x‐ray tube output) than all other scanners, but the highest reported EAK. A few months after acquiring the measurements for the Discovery scanner, its x‐ray tube was replaced due to it failing daily QC for BMD bias outside of allowable limits. EAK measurements on the newly installed tube showed a large increase of ∼50% from its values just prior to the failure (new EAK was ∼40% higher than the values from this study). These increased values brought the Discovery system roughly in line with the measured EAK from the average of the three Horizon scanners. Reported EAK values also then fell much more in line with measured EAK on the Discovery system. It is noteworthy that reported EAK values were never observed to change at any point for any scanner, despite instances of service and x‐ray tube replacements. It seems the reported EAK values are simply based on scanner platform, scan mode, and scan length—they do not change otherwise. As a result, how closely the reported EAK matches with the true output of the system will likely vary significantly across different scanners and times. While it may be true that the reported EAK attempts to match closely with the output of a fresh x‐ray tube, just among the three Horizon scanners assessed in this study the variability in tube outputs was reasonably large and was not clearly associated with x‐ray tube age or performance status.

For general x‐ray and fluoroscopy applications, the typical regulations at the federal and state levels in the United States require that vendor‐reported radiation output (EAK, DAP, or kerma‐area‐product, KAP) be within ±35% of what is measured.^21^ There is currently not a similar requirement for DXA in the United States; however, our results show that many of the reported EAK values in this study would not fit within such requirements. Even when correcting the Horizon reported EAK values as discussed above, or including backscatter in the measured EAK data, there would still be instances of measured versus reported EAK differences beyond the ±35% threshold. The overall results suggest that the current algorithm‐based methods for vendor‐reported EAK may not be sufficient. An in situ measurement with an actual dose sensor as is used on general x‐ray and fluoroscopy equipment may become necessary if future regulations require that measured and reported EAK differences be within certain thresholds for DXA equipment.

The differences in reported EAK across scan modes could be well modeled based on changes in acquisition conditions. For example, Fast mode had reported EAK values that were consistently 50% of those for Array mode (Table 5). When referring to Table 2 this change was due simply to the 50% decrease in total scan time (doubling in scan speed) for Fast mode relative to Array mode. There was not any collimation change between Fast and Array modes, so the reported EAK must then be 50% less for Fast mode. HD mode and Array mode showed equal reported EAK for the Discovery scanner. Based on the discussion outlined above, we believe the Horizon scanners should have had the same comparison for HD and Array modes despite being different in our data here. A similar type of analysis using Table 2 helps to confirm this assessment. HD mode scans took twice as long as Array mode based on a 50% reduced scan speed, yet they also utilized a collimation width that is 50% narrower relative to Array mode. Overall, this combination of changing acquisition conditions should make reported EAK equal between HD and Array modes. When combining the changing scan speed and collimation between Array mode and Express mode, the change in reported EAK between the two scan modes should be close to a factor of 2.75. This is roughly halfway between the ratios calculated for Horizon (2.64) and Discovery (2.86) scanners. Note that the nominal collimation settings are different between Horizon and Discovery scanners (Table 2), but the scan field sizes at the patient skin surface were the same. Both the collimator length ratios and collimator slit width ratios are equal (∼1.3) when comparing Discovery to Horizon. So, the collimator is likely placed at a different location relative to the x‐ray source on Discovery scanners but the different collimation settings ensure this does not affect scan field size or EAK relative to Horizon scanners.

This study had limitations that should be addressed. While we were able to compare both measured and reported EAK across several different DXA scanners, they were all from the same vendor. It would be interesting to see if other vendors’ DXA systems maintained similar results as those shown here. Additionally, the true EAK values were not known for any scanner or scan mode. Our measurements attempted to produce the most accurate reflection of EAK possible, but due to the narrow x‐ray fan beam geometry they may still have had errors. The dose sensors used in this study are designed to be uniformly and completely exposed to radiation, which is not possible on modern DXA scanner setups. These types of measurement issues have been discussed before in previous work,^22^, ^23^ though we are not aware of any prior studies attempting to address these limitations specifically for DXA exposure/dose measurements.

The primary issue of concern is the potential non‐uniform response in the ion chamber or solid‐state detector as the narrow fan beam of radiation impinges upon and passes across it.^22^ Especially for the ion chambers, the response and sensitivity may differ when the middle region at full diameter is exposed relative to the outer regions at narrower diameters as the beam scans across the chamber. More detailed analysis of the dosimetry device response to narrow x‐ray beams as outlined previously^22^, ^23^ is worthy of future work. It may also be possible to calibrate CR plate pixel intensities to infer exposure/dose in an effort to estimate EAK from the full length scans of the DXA systems.^12^, ^19^, ^24^ A DAP meter could also provide a more accurate assessment of EAK and/or DAP if set up appropriately. Despite the study's limitations, our results showed differences between scanners and platforms of a single vendor, as well as inconsistencies between measured and reported EAK that were not associated with the measurement conditions utilized.

Finally, Table 1 shows that the nominal entrance skin doses outlined in vendor documentation suggest that entrance dose (and seemingly, EAK) should be the same for all the DXA scanners assessed in this study—even Horizon compared to Discovery, but the reported EAK values were higher for Discovery than Horizon, and were also higher than the nominal entrance skin dose values outlined in Table 1. We also found a relatively wide range of measured EAK values across the scanners tested. We are not aware of any reason why the published nominal entrance skin dose estimates would be the same, and yet reported EAK different, between Horizon and Discovery systems. Nor are we aware of any reason why the nominal entrance dose values are all lower than the EAK values derived from vendor‐reported DAP and scan field size. The ratios between vendor‐reported EAK (Table 5) and vendor‐outlined nominal entrance dose (Table 1) were not consistent across the different scanners or scan modes, with EAK being from 30% to 83% higher relative to entrance dose. Based on our measurements, the scan field sizes should be nominally the same for Horizon and Discovery and we have x‐ray tube output data showing that Discovery can be much lower or very similar to that from Horizon scanners. Yet the reported EAK for Discovery was consistently different from Horizon and the ratios between the two scanners reported EAK values were not consistent among all scan modes. All of these results impress upon the idea that verifying tube output at baseline and over time is an important aspect of DXA quality assurance. Ideally the comparison to reported EAK is useful to understand any issue of tube output relative to the vendor expected value. However, as noted, future work may be needed to understand the algorithms vendors use to establish EAK values for such a comparison.

## 5 CONCLUSION

EAK can be measured on DXA scanners with a variety of dose sensors without causing relevant bias or uncertainty in the measurements. This approach offers a simple method for estimating doses by requiring only a periodic measurement to ensure stability. EAK measurements also have negligible dependence on scan length. This enables flexibility around both dose sensor and lengths of scans used for EAK measurements, as not all sensor designs match with the full range of scan lengths achievable with DXA. Reported EAK was consistent among scanners of the same platform, but measured EAK varied significantly from scanner to scanner and may also change over time. As a result, measured and vendor‐reported EAK may not always be comparable. This reinforces the importance of establishing a QC program that includes a dose metric such as EAK, along with baseline measurements so that longitudinal changes can be tracked, to ensure optimal DXA equipment performance for clinical applications.

## CONFLICT OF INTEREST

The authors declare no conflict of interest.

## AUTHOR CONTRIBUTIONS

Matthew A. Thomas and Jorge E. Jimenez contributed to the project design, made all the measurements at MD Anderson, performed the data analysis, created the figures, and wrote the manuscript. Samuel J. Fahrenholtz and Kushnood A. Hamdani made all the measurements at Mayo Clinic and edited the manuscript. William D. Erwin initiated the project, contributed to its design, oversaw the general progress, and edited the manuscript.
